# Anti-Inflammatory Activities of Betulinic Acid: A Review

**DOI:** 10.3389/fphar.2022.883857

**Published:** 2022-05-23

**Authors:** José Fernando Oliveira-Costa, Cássio Santana Meira, Maria Vitória Gomes das Neves, Bruna Padilha Zurita Claro Dos Reis, Milena Botelho Pereira Soares

**Affiliations:** ^1^ Center for Infusions and Specialized Medicines of Bahia, Bahia State Health Department, Salvador, Brazil; ^2^ SENAI Institute of Innovation in Health Advanced Systems (ISI SAS), University Center SENAI/CIMATEC, Salvador, Brazil; ^3^ Gonçalo Moniz Institute, Oswaldo Cruz Foundation (FIOCRUZ), Salvador, Brazil

**Keywords:** betulinic acid, anti-inflammatory activity, terpenoids, immunomodulation, inflammation

## Abstract

Inflammatory diseases have a high prevalence and has become of great interest due to the increase in life expectancy and the costs to the health care system worldwide. Chronic diseases require long-term treatment frequently using corticosteroids and non-steroidal anti-inflammatory drugs, which are associated with diverse side effects and risk of toxicity. Betulinic acid, a lupane-type pentacyclic triterpene, is a potential lead compound for the development of new anti-inflammatory treatments, and a large number of derivatives have been produced and tested. The potential of betulinic acid and its derivatives has been shown in a number of pre-clinical studies using different experimental models. Moreover, several molecular mechanisms of action have also been described. Here we reviewed the potential use of betulinic acid as a promissory lead compound with anti-inflammatory activity and the perspectives for its use in the treatment of inflammatory conditions.

## Introduction

**FIGURE 1:**
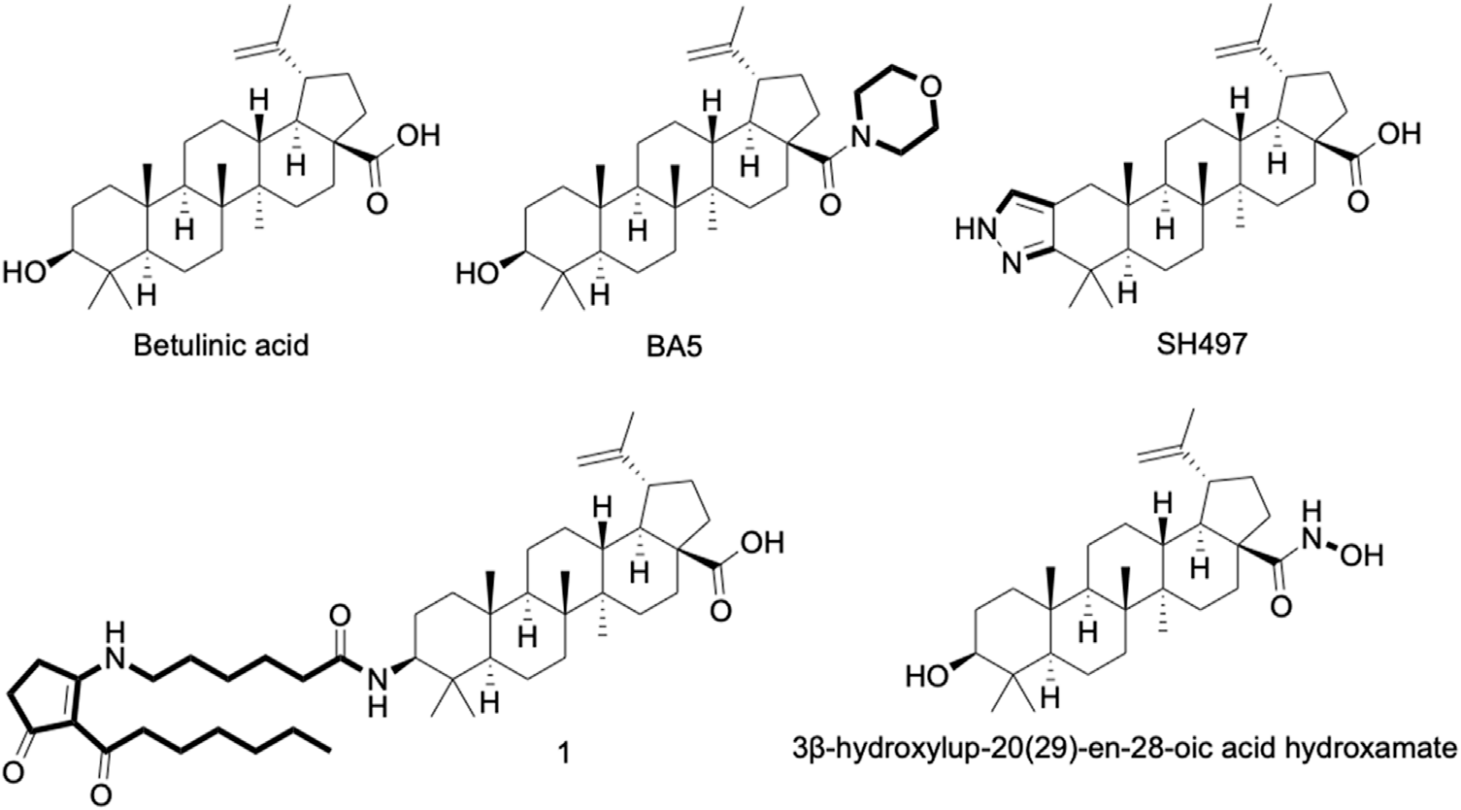
Chemical structure of betulinic acid (BA) and its derivatives with anti-inflammatory activity well-known. 1 = 3-Deoxy-3β-((6-(2-heptanoyl-3-oxocyclopent-1-en-1-yl)amino)hexanamido))betulinic acid.

Terpenes are secondary metabolites produced naturally in plants, as a result of interactions with the environment. They are also found in mosses, algae, and lichen, and some can be also found in mammals (Zhou and Pichersky, 2020). In general, terpenes have been a valuable source of medical discoveries. A promising representative of the class is betulinic acid (BA) (Figure 1), a lupane type pentacyclic triterpene, described for the first time in 1917 (Traubenberg et al., 1917).

BA is normally obtained from plant sources, mainly from *Betula* species (Hajati et al., 2018). However, the purification of BA from natural sources is a time-consuming and not environmentally friendly process that results in a low yield, making extraction methods unfeasible for a large-scale production (Eckerman and Ekman, 1985). To solve this problem, alternatively routes to obtain BA were developed, such as chemical synthesis, biotransformation using fungus cultures, and metabolic engineering biosynthetic pathways in microorganisms (Dubey and Goel, 2013; Czarnotta et al., 2017).

BA has several biological activities already described, such as diuretic, antimicrobial, antiviral, antidiabetic, antiparasitic, immunomodulatory, and anticancer activities (de-Sá et al., 2009; Oliveira-Costa et al., 2014; Jiang et al., 2021). The anticancer activity of BA is considered to be promising, since it was shown to be cytotoxic against various types of cancer cells and caused the inhibition of tumor growth in xenograft mouse models (Jiang et al., 2021).

The immunomodulatory activity of betulinic acid is also considered promising, due to its capacity to modulate several cell types of the immune system, such as macrophages and lymphocytes, and its anti-inflammatory activity was shown in different models of inflammation (Oliveira-Costa et al., 2014; Meira et al., 2017; Ou et al., 2019). In this context, this review aims to describe the main findings and mechanisms of action related to the anti-inflammatory activity of BA.

### Inflammation and Natural Products

Inflammation plays an important role in resolving imbalances in the body’s homeostasis and is essential for the repair, remodeling, and renewal of different tissues under a variety of harmful conditions (Grivennikov et al., 2010). It is considered the first line of defense that protects the host against infections caused by a number of pathogens, such as bacteria, fungi, and viruses (Rock et al., 2010). In addition, other non-infectious stimuli can also trigger inflammation, such as damaged cells, chemical agents, physical injury, burns, radiation, frostbite, ischemia, and reperfusion (Chen and Nuñez, 2010; Rock et al., 2010). However, inflammation can become uncontrolled, potentially causing a range of dysfunctions, including autoimmune diseases, asthma, inflammatory bowel diseases, cardiovascular complications, among others (Sultana and Saify, 2012; Oyebanji et al., 2014; Arulselvan et al., 2016).

The inflammatory process is complex, involving cellular events, such as the migration of leukocytes (neutrophils, monocytes, basophils and eosinophils) (Huether and Mccange, 2015), the extravasation of plasma and fluids to the site of inflammation, and release of specific mediators and other signaling molecules (including eicosanoids, leukotrienes, histamine, cytokines, chemokines, platelet activating factor, free radicals derived from oxygen and nitrogen, and serotonin) by endothelial cells, resident leukocytes such as macrophages, mast cells, and dendritic cells, or by newly recruited cells (Anwikar and Bhitre, 2010).

Classically, treatment of inflammation is done with non-steroidal anti-inflammatory drugs and steroids, in an attempt to improve the patient’s symptoms (Gupta and Dubois, 2002). Although effective in treating inflammation to varying degrees, both drug classes have a range of undesirable side effects, such as gastrointestinal disturbances, cardiac changes, renal toxicity, hypertension, type 2 diabetes, visceral obesity, and atherosclerosis (Chapmann et al., 2013; Harirforoosh et al., 2013; Sostres et al., 2013).

Thus, the development of new substances with anti-inflammatory activity is of great importance for clinical use, in order to obtain alternative treatments with efficacy and fewer adverse effects. A widely used strategy has been the prospection of molecules with anti-inflammatory potential in medicinal plants, given that plants have a vast diversity of molecules that are still largely unknown (Newman and Cragg, 2016). The pharmacological activities of natural products have been widely reported in the literature, including anti-inflammatory activity, since the discovery of salicylic acid by Stone in 1763. Subsequently, a large number of molecules with anti-inflammatory activity has been identified, many of them from plants used in folk medicine, and others due to random bioprospection.

Among the natural compounds with anti-inflammatory activity, betulinic acid was reported for the first time by Recio et al., in 1995. Subsequently, its pharmacological properties continued to be widely reported in the scientific literature due to its diverse biological activities, in addition to anti-inflammatory actions in different models (Nader, 2012; Oliveira-Costa et al, 2014).

### *In Vitro* Anti-Inflammatory Activity of BA

**FIGURE 2:**
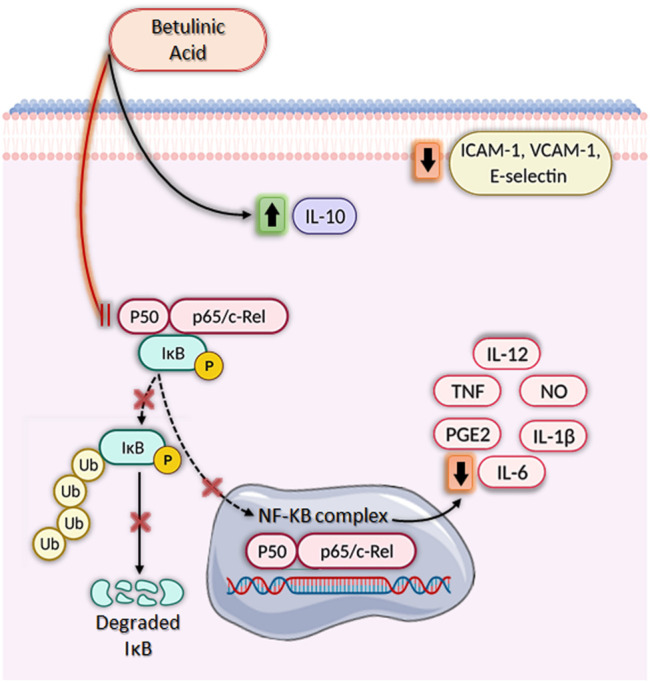
Immunomodulatory activities of betulinic acid *in vitro*. Betulinic acid (BA) has a broad-spectrum anti-inflammatory activity, significantly increasing IL-10 production, decreasing ICAM-1, VCAM-1, and E-selectin expression and inhibiting nuclear factor kappa-light-chain-enhancer of activated B cells (NF-κB), leading to downregulation of several pro-inflammatory genes. BA blocks the NF-κB signaling pathway by inhibiting IκB phosphorylation and degradation through ubiquitination via the proteasome degradation machinery. As a result, NF-κB is not activated and does not translocate from the cytoplasm to the nucleus, impeding the transcription of pro-inflammatory mediators such as IL-1β, IL-6, IL-12, NO, PGE2, and TNF. Created with BioRender.com.

BA has been shown to modulate the activity of several cell types and molecules involved in the inflammatory response (Table 1; Figure 2). A critical inflammatory mediator is nitric oxide (NO), produced from l-arginine by the action of the enzyme inducible nitric oxide synthase (iNOS) (Förstermann and Sessa, 2012). If produced in excess, NO leads to the development of various inflammatory diseases, such as arthritis, inflammatory bowel disease, and multiple sclerosis (Sharma et al., 2007). Thus, inhibition of iNOS and/or NO production can be assessed to evaluate anti-inflammatory properties. Interestingly, several *in vitro* studies have shown that BA can inhibit the production of NO, mainly in macrophages cultures stimulated with bacterial lipopolysaccharide (LPS) and/or interferon gamma (IFN-ɣ) (Yun et al., 2003; Blaževski et al., 2013; Oliveira-Costa et al., 2014; Jingbo et al., 2015; Kim et al., 2016; Meira et al., 2017; Ryu et al., 2020).

**TABLE 1:** *In vitro* anti-inflammatory activity of betulinic acid.

Reference	Main result
Dustan et al., 1998	BA inhibited bovine prostaglandin synthase
Yun et al., 2003	BA decreased NO and COX-2 levels in RAW 264.7 macrophages
Viji et al., 2011	BA decreased IL-6 production through modulation of NF-κB pathway
Yoon et al., 2010	BA significantly decreased TNF-induced ICAM-1, VCAM-1 and E-selectin expression levels. In addition, inhibited NF-κB activation
Blaževski et al., 2013	BA inhibited IL-17 and IFN-γ production in a concentration dependent manner in lymphocytes cultures. In addition, significantly increased ROS generation, and suppressed NO generation in macrophages cultures
Costa et al., 2014	BA inhibited IL-6, NO and TNF and increased of IL-10 production by peritoneal macrophages
Jalil et al., 2015	BA showed an IC_50_ of 2.59 in PGE_2_ production
Jingbo et al., 2015	BA inhibited IL-1β-induced MMP-1, MMP-3, MMP-13, PGE_2_ and NO production and NF-κB activation. In addition, BA was found to activate PPAR-γ in human osteoarthritis chondrocytes
Kim et al., 2016	Inhibition of pro-inflammatory mediators such as PGE_2_, NO, IL-1β, IL-6, IL-12, and TNF in LPS-induced RAW 264.7 cells and suppression of NF-κB signaling pathway. In addition, BA induced HO-1 induction via Nrf2 translocation
Meira et al., 2017	BA promoted a reduction of NO and TNF production and NF-κB activity and increased IL-10 production in macrophages. In addition, inhibited lymphoproliferation, IL-2, IL-4, IL-6, IL-17A and IFNγ and also increased IL-10 production in lymphocytes cultures activated with Con A
Li et al., 2019	BA treatment suppressed the migration, invasion and reorganization of the actin cytoskeleton of RA FLSs. In addition, we found that the mRNA expression of IL-1β, IL-6, IL-8, and IL-17A were markedly down-regulated by treatment with BA via NF-κB pathway
Ryu et al., 2000	Inhibition of NO production by RAW 264.7 macrophages

BA, betulinic acid; Con A, concanavalin A; COX-2: cyclooxygenase-2, FLS: fibroblast-like synoviocytes; HO-1, heme oxygenase-1; ICAM-1, intercellular adhesion molecule-1; IC_50_, inhibitory concentration of 50%; IL-1β, Interleukin-1, beta; IL-2, Interleukin-2; IL-6, Interleukin-6; IL-10, Interleukin-10; IL-12, Interleukin-12; IL-17A, Interleukin-17A; lipopolysaccharide; MMP1, Matrix metalloproteinase-1; MMP3, Matrix metalloproteinase-3; MMP13, Matrix metalloproteinase-13; NF-κB, Nuclear factor kappa-light-chain-enhancer of activated B cells; NO, nitric oxide; PPAR-γ, peroxisome proliferator-activated receptor gamma; PGE_2_, prostaglandin E_2_; RA, rheumatoid arthritis; TNF, tumor necrosis factor; VCAM-1, vascular cell adhesion molecule-1.

Furthermore, BA also inhibits cyclooxygenase-2 (COX-2) activity and, therefore, decrease prostaglandin E_2_ (PGE_2_) synthesis (Dustan et al., 1998; Jalil et al., 2015; Jingbo et al., 2015; Kim et al., 2016). PGE_2_ is responsible for inflammatory symptoms, such as fever, pain, and platelet aggregation, and thus measuring the reduction of PGE_2_ production is an attractive strategy for the discovery of anti-inflammatory drugs (Park et al., 2006). The production of critical pro-inflammatory cytokines, such as IL-1β, IL-6, IL-8, IL-12, and TNF, is also decreased by BA treatment (Oliveira-Costa et al., 2014; Kim et al., 2016; Meira et al., 2017; Li et al., 2019). Most of these effects are related to the inhibition of nuclear factor kappa-light-chain-enhancer of activated B cells (NF-κB), a transcription factor involved in the regulation of several pro-inflammatory genes (Figure 1) (Yoon et al., 2010; Jingbo et al., 2015; Kim et al., 2016; Meira et al., 2017; Li et al., 2019). Inhibition of NF-κB activation by BA also decreases the expression of adhesion molecules, such as ICAM-1, VCAM-1, and E-selectin, in endothelial cells, which may have implications for the treatment of vascular inflammation (Yoon et al., 2010). Interestingly, the effects of BA on NF-κB pathway is potentiated by the presence of inhibitors of mitogen activated protein kinases (MAPK), such as SB203580 (p38 inhibitor) and PD98059 (extracellular signal-regulated kinase inhibitor) (Viji et al., 2011). Kim et al. (2016) also demonstrated that induction of HO-1 enzyme activity is associated with the anti-inflammatory effect of BA, since SnPP, an inhibitor of HO-1, promoted a partial reversal of BA’s effect on NF-κB activity, as well as on IL-1β, IL-6, NO, PGE_2_, TNF, IL-1β, and IL-6 production. In addition to the reduced production of pro-inflammatory molecules, BA also increased the amount of IL-10, a well-known anti-inflammatory cytokine (Oliveira-Costa et al., 2014; Meira et al., 2017). Lastly, BA also modulated lymphocyte function through the inhibition of lymphoproliferation, decreasing the production of pro-inflammatory cytokines, such as IL-2, IL-6, IL-17, and IFN-γ (Blaževski et al., 2013; Meira et al., 2017).

### Anti-Inflammatory Activity of BA *in Vivo*

The anti-inflammatory effects of BA have also been validated in various animal models (Table 2). The initial work by Recio et al. (1995) tested BA, isolated from the leaves of *Diospyros leucomelas*, in three mouse model of inflammation: 12-O-tetradecanoylphorbol acetate (TPA)-induced ear edema, carrageenan-induced paw edema and ethyl phenylpropiolate (EPP)-induced ear edema, all models using Swiss mice. BA, administered topically at 0.5 mg/kg, induced a reduction in TPA edema and EEP edema. In addition, BA administered orally at 100 mg/kg, alsos promoted a reduction in paw edema induced by carrageenan (Recio et al., 1995). Further studies confirmed the anti-inflammatory activity of BA in carrageenan-induced edema, in doses ranging from 10 to 100 mg/kg, mainly by oral route (Mukherjee et al., 1997; Tsai et al., 2011; Oyebanji et al., 2014; Armah et al., 2015; Ou et al., 2019). Importantly, when administered by intraperitoneal route, BA also reduced carrageenan-induced paw edema in Wistar rats (Oyebanji et al., 2014). In addition, Oyebanji et al. (2014) observed a reduction in carrageenan-induced-pulmonary edema in Wistar rats treated intraperitoneally with BA at 10, 20 or 40 mg/kg.

**TABLE 2:** *In vivo* immunomodulatory activity of betulinic acid.

References	Route/dose	Model	Main result
Recio et al. (1995)	Orally/100 mg/kg or 0.5 mg/ear	Carrageenan-induced paw edema, TPA-induced mouse ear edema and EPP-induced mouse ear edema in Swiss mice	BA promoted inflammation reduction in all models specially in TPA-induced mouse ear edema
Mukherjee et al. (1997)	Orally/50 or 100 mg/kg	Carrageenan-induced paw edema or serotonin-induced paw edema in Wistar rats	Reduction of paw edema in both models
Huguet et al. (2000)	0.5 mg/ear	Mezerein-, 12-deoxyphorbol-13-tetradecanoate-induced mouse ear edema or bryostatin 1-induced mouse ear edema	Reduction of ear edema in both models
Viji et al. (2011)	I.P/20 mg/kg	Endotoxic shock-induced by LPS in Swiss mice	A significantly reduction sepsis-induced mortality and lung injury. In addition, decreased PGE_2_ production and MPO activity
Tsai et al. (2011)	Orally/10, 20 or 40 mg/kg	Carrageenan-induced paw edema in ICR mice	BA reduced paw edema, COX-2, NO, IL-1β, TNF. and MDA levels. In addition, BA treatment increased antioxidant enzyme activities (SOD, GPx and GRd)
Nader et al. (2012)	Orally/25 mg/kg	Lipopolysaccharide-induced lung inflammation in Sprague-Dawley rats	BA reduced lung inflammation by inhibited cell recruitment, TNF, NO, and, TGF-β1 expression. In addition, promoted activation of antioxidant system by attenuate MDA production and increase GSH and SOD activity
Costa et al., 2014	I.P./33 or 67 mg/kg	Endotoxic shock-induced by LPS in BALB/c mice or C57BL/6 IL-10 −/− mice	BA protected mice against a lethal LPS challenge through IL-10 production
Oyebanji et al. (2014)	I.P./10, 20 or 40 mg/kg	Carrageenan-induced paw edema and carrageenan-induced-pulmonary edema in Wistar rats	BA significantly reduced carrageenan-induced paw edema by 11.0, 45.7, 68.6% or pulmonary edema by 25.6, 29.2 and 45.1% at doses of 10, 20 and 40 mg/kg respectively
Lingaraju et al. (2015a)	I.P./3, 10 or 30 mg/kg	Endotoxic shock-induced by cecal ligation and puncture in Swiss mice	BA significantly reduced sepsis-induced mortality and lung injury. In addition, decreased IL-6, TNF, ICAM-1, MCP-1, MPO, MMP-9 and NF-κB activity
Lingaraju et al. (2015b)	I.P./3, 10 or 30 mg/kg	Sepsis-induced by cecal ligation and puncture surgical procedure in Swiss mice	BA reduced sepsis-induced lung injury, at least in part, through its ability to balance oxidant-antioxidant status and to inhibit neutrophil infiltration and attenuated histopathologic changes
Armah et al. (2015)	Orally/10, 30 or 100 mg/kg	Carrageenan-induced paw edema in chicken	Reduction of paw edema
Kalra et al. (2018)	Orally/3, 10 or 30 mg/kg	Dextran sulfate sodium-induced colitis in Swiss mice	BA prevented diarrhea; bleeding and colonic pathological changes induced by DSS. Further, BA decreased oxidative stress and inflammatory factors such as MMP-9 and PGE_2_
Huimin et al. (2019)	Orally/20 or 40 mg/kg	Freund’s complete adjuvant-induced arthritis in rats	BA can significantly inhibit the arthritis index, improve joint pathology, reduce toe swelling, improve blood rheology, improve synovial cell apoptosis, and restore related cytokine negative regulation of ROCK/NF-κB signaling pathways
Li et al. (2019)	I.P./20 mg/kg	Arthritis-induced by type II collagen in DBA/1 mice	BA attenuated synovial inflammation and joint destruction in mice with CIA
Ou et al., 2019	Orally/2.5, 5 or 40 mg/kg	Carrageenan-induced paw edema in Kunming mice	BA reduced paw edema, neutrophil infiltration and also IL-1α, IL-1β, IL-5, IL-6, GM-CSF, KC, MCP-1, and PGE_2_ levels. In addition, decreased the expression of COX-2 protein, and reduced the phosphorylation of JNK, p38 and ERK1/2
Zhou et al. (2021)	I.P./1, 5 or 10 mg/kg	Acute pancreatitis-induced by ceruein in C57BL/6 mice	BA attenuated pancreatitis through NF-κB pathway

CIA, collagen-induced arthritis; COX-2, cyclooxygenase-2; ERK, extracellular signal-regulated kinase; EPP, ethyl phenylpropiolate; GPx, glutathione peroxidase; GSH, glutathione; JNK, c-Jun N-terminal kinase; ICAM-1, Intercellular Adhesion Molecule 1; IL-6, Interleukin-6; IL-10, Interleukin-10; I.P., intraperitoneal route; LPS, lipopolysaccharide; MCP-1, monocyte chemoattractant protein-1; MDA, malondialdehyde; MMP9, Matrix metalloproteinase-9; MPO, myeloperoxidase; NF-κB, Nuclear factor kappa-light-chain-enhancer of activated B cells; NO, nitrite; PGE_2_, prostaglandin E_2_; SOD, superoxide dismutase; TNF, tumor necrosis factor; T.A., topical application; TGFβ1, Transforming growth factor beta 1; TPA, 12-O-tetradecanoyl-phorbol-13-acetate.

**FIGURE 3:**
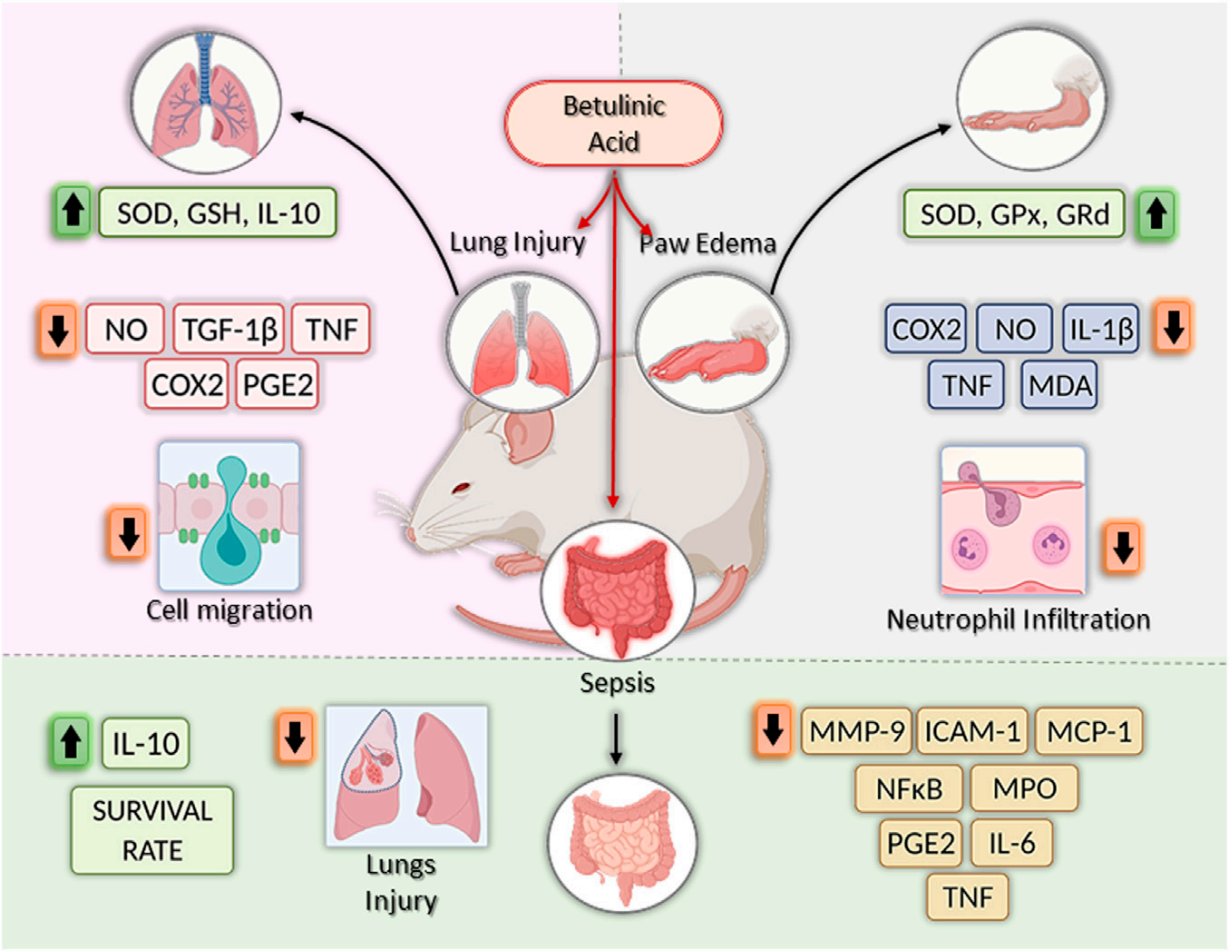
Effects of betulinic acid *in vivo* models. Betulinic acid (BA) reduced lung inflammation by increasing levels of GSH, SOD and IL-10, by inhibiting the expression of proinflammatory mediators, such as NO, TGF-β1, TNF, COX-2, and PGE2, and by reducing cell migration to the lesion site. In the paw edema models, BA treatment increased the activities of antioxidant enzymes (SOD, GPx and GRd), reduced COX-2, IL-1β, NO, TNF, MDA, and TNF, as well as neutrophil infiltration, leading to the reduction of swelling in the paw. Finally, in sepsis models, BA treatment increase survival rate by decreasing levels of MMP-9, ICAM-1, MCP-1, PGE2, IL-6, and TNF, reducing lung injury and decreasing MPO and NF-κB activity. In addition, increase IL-10 production. Created with BioRender.com.

The main mechanism associated with carrageenan-induced inflammation is well characterized and involves the reduction of inflammatory mediators such as COX-2, Il-1β, NO, PGE_2_, and TNF (Salvemini et al., 1996; Tsai et al., 2011). In addition, the inhibition of antioxidant enzymes, lipid peroxidation, and production of free radicals, such as hydrogen peroxide, superoxide, and hydroxyl radical in the liver, are common features in carrageenan-induced inflammation related to cell injury (Cuzzocrea et al., 1999). Interestingly, treatment with BA decreased the production of the inflammatory mediators described above at the inflammation site and increased enzyme activity of superoxide dismutase (SOD), glutathione peroxidase (GPx), and glutathione reductase (GRd) in the liver (Tsai et al., 2011; Ou et al., 2019). Moreover, BA decreased malondialdehyde (MDA) levels, a key mediator of oxidative stress and widely used as a marker of free radical mediated lipid peroxidation injury, at the inflammation site (Tsai et al., 2011). Lastly, Ou et al. (2019) provided evidence that BA downregulates MAPK signaling pathways (ERK1/2, JNK, and p38) in the paw edema tissue, which, in part, explains the inhibition of cytokine production (IL-1β and TNF), COX-2 expression, and PGE_2_ production (Figure 3).

Another model in which BA was well explored is sepsis, which can be induced by LPS injection or cecal ligation and puncture (CLP), both models useful in preclinical trials to screen new anti-inflammatory agents (Viji et al., 2010; Oliveira-Costa et al, 2014; Lingaraju et al., 2015a; Lingaraju et al., 2015b). Initially, BA (20 mg/kg) was tested by intraperitoneal route, in a model of endotoxic shock-induced by LPS in Swiss mice (Viji et al., 2010). BA increased the survival rate of animals, and 40% of the mice pretreated with BA remained alive after the observation time (7 days). In addition, BA reduced PGE_2_ production and myeloperoxidase (MPO) activity, an index of neutrophil infiltration, in liver and lung tissue (Viji et al., 2010). Using a similar model, Oliveira-Costa et al. (2014) using a higher dose of BA (67 mg/kg) observed a more pronounced protective effect (100% survival rate) in BALB/c mice treated intraperitoneally with BA and challenged with a lethal dose of LPS. Moreover, the protective effect was accompanied by a reduction of the pro-inflammatory cytokine TNF and an increase in the anti-inflammatory cytokine IL-10 in the sera of BA-treated mice, as well as in cultures of peritoneal macrophages obtained from animals treated with BA (67 mg/kg). Using IL-10 deficient C57BL/6 mice, the role of IL-10 in the BA-induced protection against LPS challenge was confirmed, since BA did not protect mice in the absence of IL-10 (Costa et al., 2014). Since IL-10 was shown to be produced by activation of the NF-kB pathway (Saraiva and O´Garra, 2010), and due to the effect of BA in reducing NF-kB activation, the mechanisms leading to the increase in IL-10 production induced by BA still remains to be determined. BA also promoted the increase in survival rate in a sepsis model induced by CLP, in mice pretreated intraperitoneally with BA (Lingaraju et al., 2015a).

Importantly, BA prevented lung injury by decreasing the production of IL-6, MCP-1, MMP-9, TNF, and MPO activity, all these effects related to inhibition of NF-κB activation (Lingaraju et al., 2015a). Interestingly, using the same model, Lingaraju et al. (2015b) also demonstrated that BA reduced lung injury induced by sepsis, at least in part, through its ability to balance oxidant-antioxidant status and to inhibit neutrophil infiltration (Lingaraju et al., 2015b). In accordance with these data, oral administration of BA at 25 mg/kg also reduced lung inflammation induced by LPS in Sprague-Dawley rats (Nader et al., 2012).

In a model of ulcerative colitis (UC) caused by dextran sulfate sodium (DSS) (Matos et al., 2013), BA decreased oxidative stress, production of some inflammatory factors, and visceral pain, fundamental aspect to intestinal bowel diseases (IBD) therapy (Kalra et al., 2018). Thus, while control group had loss in body weight, loose stool consistency, and gross rectal bleeding at end of experiment, the BA oral administration showed improvement of symptoms. BA (at 10 and 30 mg/kg) was able to decrease the bleeding score and augment the stool consistency (at 30 mg/kg), when compared to the control. The disease activity index (DAI) was significantly lower in BA-treated group (10 and 30 mg/kg), if compared to DSS group. BA also reduced colon shortening, observed in the DSS control group. If considered oxidative stress markers, BA was able to reduce nitrite and serum lipid hydroperoxide levels. While the DSS group had colon malondialdehyde (MDA), nitrite levels and serum lipid hydroperoxide increased, to the BA group, this oxidative stress markers were reduced. While in the DSS group was observed an anti-oxidants like superoxide dismutase (SOD), catalase and reduced glutathione (GSH) reduction, BA treatment normalized these parameters, showing protection. BA treatment (30 mg/kg) also inhibited MPO, MMP-9, and PGE_2_. Moreover, histopathological analyses showed that BA ameliorates mucosa destruction and inflammatory changes, with improving of histological aspects (Kalra et al., 2018).

BA acts on acetic acid-induced writhing (Mohammad et al., 2012; Qu et al., 2015) and mustard oil-induced visceral nociception (Laird et al., 2001), producing an effect comparable to that of etoricoxib, if administered 1 hour before challenge, inhibiting the writhing response and suppressing pain response, in a dose-dependent way (Kalra et al., 2018). BA at 10 and 30 mg/kg was able to protect mice against MO-induced plasma extravasation in colon and death, showing to be an effective molecule in this model of inflammation and analgesia. BA have protective effects in DSS-induced colitis and antinociceptive capacity in an experimental visceral pain model, being a promising agent to the IBD treatment (Kalra et al., 2018).

BA showed a toxicity at 30 mg/kg dose, inducing the increase in ALT levels, indicating the use of lower doses for *in vivo* experiments, as performed by Zhou (2021), which tested BA in a model using cerulein-induced acute pancreatitis (AP). Pre-treatment with BA is able to decrease pancreatic damage, in analyses observing reduction in acinar cell death, pancreatic edema, inflammatory cell infiltration and pancreatic myeloperoxidase (MPO) activity, reflecting reduction of neutrophil sequestration. Prior administration of BA reduced the levels of amylase and lipase, increased in acute pancreatitis. Lung injury, a common manifestation in acute pancreatitis, was reduced by pretreatment with BA in the acute pancreatitis model. Additionally, BA administration reduced the expression of IL-1, IL-6, and TNF mRNA and proteins in the pancreas, increased in animals with AP. COX-2 mRNA expression, increased in AP, was reduced by BA pretreatment during cerulein-induced pancreatitis. Moreover, prevention of cell death and production of proinflammatory cytokines by pancreatic acinar cells (PAC’s) is observed in BA-treated animals, in a dose-dependent manner. Macrophage and neutrophil pancreas infiltration were also reverted by BA treatment. Finally, in the acute pancreatitis model, BA was also shown to modulate NF-κB activation and mitogen activated protein kinase (MAPK), inhibiting Iκ-Bα degradation, NF-κB p65 translocation into the nucleus. and NF-κB binding activity, without inhibiting the phosphorylation of P38, c-JUN N-terminal kinase (JNK), and extracellular signal-regulated kinases (ERK) (Zhou et al., 2021).

Rheumatoid arthritis (RA) is an important joint disease, immune-mediated condition, with a chronic inflammation associated to progressive synovial hyperplasia and destruction of bone cartilage. Strong evidence indicates the participation of fibroblast-like synoviocytes (FLS’s) in the synovial inflammation and joint erosion as an important player in the process. Activated FLS in RA possess a tumor-like property and different biological characteristics, such as anchorage-independent growth, aggressive migration and invasion, and overexpression of pro-inflammatory cytokines. Although BA at 10 μM did not affect the cell viability of RA FLS, it was not only able to inhibit the migration of RA FLS but also suppressed its invasive ability. Moreover, BA reduced the organization of actin stress fibers and cytoskeleton score, and reduced mRNA expression of IL-1β, IL-6, IL-8, IL-17A, as well as NF-κB nuclear accumulation. Treatment *in vivo* with BA suppressed the clinical manifestations of RA, characterized by reduction of synovitis, synovial hyperplasia, and invasion into calcified cartilages and bone. These observations reinforce the potential of BA to inhibit the progress of RA (Li et al., 2019).

Finally, Huimin et al. (2019), studying the pharmacological activity of BA on adjuvant-induced arthritis model in rats, showed that this compound has protective effects. BA treatment reduced the arthritis index, improved joint pathology, reduced toe swelling, improved blood rheology, decreased synovial cell apoptosis, and normalized the production of inflammatory cytokines, also acting through the modulation of ROCK/NF-kB pathways.

### BA as a Prototype for New Anti-Inflammatory Agents

In addition to having its anti-inflammatory activity described in several experimental models, BA is also considered a promising prototype for the development of more active anti-inflammatory agents (Figure 1) (Meira et al., 2017). Structural changes in the substituents of C-3, C-20 and C-28 of BA were shown (Kim et al., 1998). Modifications in BA structure carried out in C-28 have already contributed to the optimization of compounds with anti-HIV, antitumor, anti-influenza A, and anti-herpes activities (Sun et al., 1998; Jeong et al., 1999; Baltina et al., 2003; Pavlova et al., 2003).

BA5, a semi-synthetic amide derivative of BA, showed a promising anti-inflammatory activity. Activated macrophage cultures produced less NO, TNF and NF-κB activity when incubated in the presence of BA5. Similarly, this compound showed a potent inhibitory effect in activated lymphocyte cultures, inhibiting their proliferation and IL-2 secretion in a concentration-dependent manner. In addition, BA5 showed a protective effect against a lethal dose of LPS in a mouse model of endotoxic shock and decreased edema formation in a delayed-type hypersensitivity model induced by bovine serum albumin (Meira et al., 2017). Interestingly, BA5 (1 and 10 mg/kg) given by oral administration decreased heart inflammation and fibrosis in a C57BL/6 model of chronic cardiomyopathy caused by Chagas disease (Meira et al., 2019). These effects were accompanied by a reduction of pro-inflammatory cytokines, such as IFN-γ and TNF, as well as by the increase in IL-10 production. Importantly, BA5 promoted polarization to anti-inflammatory/M2 macrophage phenotype evidenced by the increase in M2 markers, such as arginase one and chitinase-3-like protein one and a decrease in the expression of nitric oxide synthase two and proinflammatory cytokines, in Chagasic mice treated with BA5 (Meira et al., 2019).

Another promising compound is the heterocyclic ring-fused BA derivative, SH479, which showed potent anti-inflammatory effect in a model of collagen-induced arthritis, acting by inducing a shift in pathogenic Th17/Th1 response to a Th2/Treg phenotype. Moreover, an additional articular bone protection effect was seen in SH479-treated animals (Chen et al., 2017). A series of betulinic acid-azaprostanoid hybrids also showed anti-inflammatory activity in a mouse model of paw edema-induced by concanavalin A (Khlebnicova et al., 2019).

Lastly, a hydroxamate of betulinic acid prevented colon inflammation and fibrosis in TNBS- and DSS-induced inflammatory bowel disease models (Prados et al., 2020). In addition, this compound promoted a significant reduction of fibrosis markers, such as tenascin C, collagen type I alpha two chain, collagen type III alpha one chain, TIMP metallopeptidase inhibitor one and alpha smooth muscle actin, as well as inflammatory markers (F4/80+, CD3^+^, Il-1β, Ccl3), in colon tissue samples (Padros et al., 2021).

## Concluding Remarks

Betulinic acid proved to be a versatile molecule, able to modulate a number of key mediators in the inflammatory process, including COX-2, ICAM-1, IL-1β, IL-6, IL-12, MCP-1, PGE_2_, and TNF, both *in vitro* and *in vivo,* in different disease models (Figures 2, 3). Most of these effects related to inhibition of NF-kB and MAPK pathways. Importantly, BA promotes the production of IL-10, a critical anti-inflammatory mediator able to modulate several immune cell types (Saraiva and O’Garra, 2010). Moreover, BA can be produced by synthetic routes and its structural changes have generated more potent and selective derivatives, making its use as a prototype for the generation of new classes of anti-inflammatory drugs promising. In order to develop BA-based treatments, there is a need for toxicological, as well as clinical studies that will demonstrate the safety and efficacy of this compound in inflammatory and immune-mediated diseases.
